# Role of Local Infiltration of Tranexamic Acid in Reducing Blood Loss in Peritrochanteric Fracture Surgery in the Elderly Population

**DOI:** 10.5704/MOJ.1611.013

**Published:** 2016-11

**Authors:** SR Virani, AA Dahapute, I Panda, SS Bava

**Affiliations:** Department of Orthopaedics, Seth GS Medical College and KEM Hospital, Mumbai, India

## Abstract

**Introduction:** Peritrochanteric fractures are common injuries occurring in elderly patients. Surgeries for these fractures are associated with significant blood loss. Intravenous tranexamic acid has a proven track record in many orthopaedic surgeries including trauma, arthroplasty and spine surgeries.

**Objective:** To study the effect of local subfascial and intramuscular infiltration of tranexamic acid in reducing blood loss and the requirement for blood transfusion in intertrochanteric fracture surgery.

**Study Design:** Single centre prospective analytical study.

**Materials and Methods:** One hundred and thirty seven patients above 65 years of age were included in the study, divided into two groups: the intervention group received subfascial and intramuscular infiltration of 2g tranexamic acid before wound closure and the control group of alternate patients did not receive any tranexamic acid infiltration. The postoperative drain output was recorded, as well as the haemoglobin level and the patients needing blood transfusion.

**Results and Conclusions:** The preoperative and postoperative haemoglobin values were recorded. The mean preoperative haemoglobin was 10.9% and 10.8% (p=0.79) in the trial and control groups respectively. The mean postoperative haemoglobin was 9.5gm% and 9.2gm% (p=0.36) in the two groups. The total postoperative blood loss in the tranexamic acid group and the control group was 190.3ml and 204.3ml respectively (p=0.25). Ten patients (14.9%) in the intervention group and 12 patients (17.1%) in the control group required blood transfusion. We conclude that tranexamic acid does not play a significant role in reducing postoperative blood loss and blood transfusion when used locally in peritochanteric fracture surgery. However a larger double blinded study comparing various modalities of use of tranexamic acid is needed to conclusively establish its role.

## Introduction

Peritrochanteric fractures commonly occur in the elderly population^1^. Dynamic hip screw with barrel plate and cephalomedullary nails are the usual methods of internal fixation in these fractures. The former method is associated with significant exposure and dissection for application of the plate. This may be associated with significant blood loss. The elderly population with their age and diminished cardiopulmonary reserves are highly susceptible to cardiovascular decompensation in the event of blood loss ^2,3^. This might necessitate blood transfusions which are associated with a plethora of harmful effects. A low level of hemoglobin is associated with delayed wound healing and rehabilitation.

Various pharmacological options like antifibrinolytics, fibrin glue, thrombin gelatin matrix and factor VIII concenterates are being used to reduce blood loss in orthopaedic surgeries^4,5^. Tranexamic acid is a synthetic analogue of the amino acid lysine that inhibits fibrinolysis, thereby reducing blood loss. Intravenous tranexamic acid has a proven track record in many orthopaedic surgeries such as total knee arthroplasty^6-10^, total hip arthroplasty^6,11^ and spine surgery^12-14^. It is now being increasingly used in various fracture surgeries^15-19^. Recently local infiltration of tranexamic acid has been proven to be more effective and less harmful in total knee arthroplasty^20,21^. This local effect of the drug has not been studied in fracture related surgeries. We have conducted a prospective study to evaluate the effect of local injection of tranexamic acid in reducing blood loss in surgeries for intertrochanteric fractures.

## Materials and Methods

The study design was that of a single centre, single blinded prospective analytical study. This study was carried out after approval by the Institutional Ethics Committee. Patients above the age of 65 years undergoing fixation of intertrochanteric fracture with dynamic hip screw and barrel plate from September 2014 to November 2015 were considered eligible for the study.

One hundred and ninety six patients above 65 years of age were included in the study after their due consent. Patients with low preoperative platelet counts, bleeding disorders and coagulopathies were excluded, as well as patients with severe hepatorenal dysfunction and cardiopulmonary disease, and those on aspirin or NSAIDS in the week preceding surgery. After exclusion, one hundred and forty one patients were found eligible. Only those surgeries which were done within one week of trauma were included. Spinal or spinal epidural anaesthesia was used and all surgeries were performed via the standard lateral vastus splitting approach, by a total of eight orthopaedic surgeons with significant expertise in hip surgery.

The intervention group of patients received intramuscular (vastus lateralis) and subfascial infiltration of 2g tranexamic acid in the proximal lateral thigh before closure. The intramuscular infiltration was mainly concentrated where the vastus lateralis is divided to gain entry on to the lateral surface of proximal femur. No infiltration was given around the fracture site or in the hip joint. The control group received no such intervention. The postoperative drain output was recorded, as well as the haemoglobin at the end of the third day and the patients needing blood transfusion. All patients were mobilized by touch weight bearing with walker as per our departmental protocol from the second postoperative day.

Four more patients were excluded before analysis as the drain did not work in two patients and was pulled out accidentally during transfer in one patient. One patient was moved to respiratory intensive care for pneumonia and hence the drain output could not be recorded by our protocol. The final number of patients available for analysis were one hundred and thirty seven with 70 in the control group and 67 in the trial or intervention group.

Although we looked for clinical evidence of pulmonary embolism or deep vein thrombosis, we did not perform any haematological or radiological investigation to assess thrombosis unless indicated clinically. Only one patient developed clinically evident deep vein thrombosis on the 5th postoperative day, confirmed by a venous Doppler. This patient, however was a part of the control group.

All data were tabulated and analysed for differences in the above mentioned end point parameters. All the collected data were entered in Microsoft Excel sheet, and then transferred to SPSS version 17.0 software for statistical analysis. Quantitative data was presented as mean and standard deviation and compared using student’s t test. A p-value of less than 0.05 was considered significant.

## Result

The mean age of the patients in the intervention group was 67 years and 69.1 years in the control group (p>0.05). Sixty-two percent of the patients in the trial group were females as opposed to 61% in the intervention group. The mean duration of the surgery was 51.2 minutes in the group receiving tranexamic acid as opposed to 49.4 minutes in the control group. (Table I)

**Table I:** Patient Demographics

Variable	Intervention group (n=67)	Control group (n=70)
Age (years)	67	69.1
Gender (females)	42/67 (62%)	43/70 (61%)
Side (left/right)	45/22	41/29
Surgical duration	51.2 minutes	49.4 minutes
Preoperative haemoglobin	10.9 (± 2.2)	10.8 (± 2.1)
Postoperative haemoglobin	9.5 (± 1.8)	9.2 (± 2.0)

**Fig. 1:**
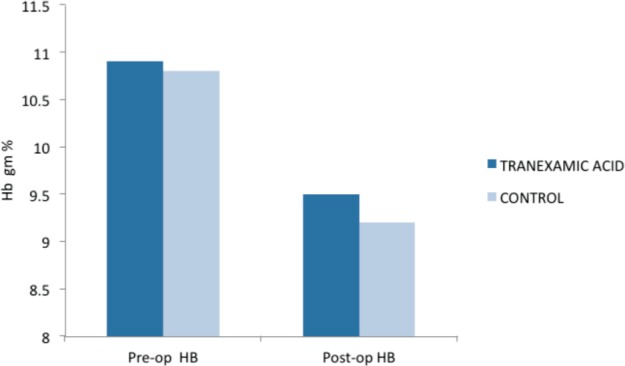
Drop in haemoglobin in both trial and control groups postoperatively.

The preoperative and postoperative haemoglobin values were recorded. The mean preoperative haemoglobin 10.9 gm% in the group receiving tranexamic acid as compared to 10.8 gm% in the group that did not receive any intervention(p=0.79). The mean postoperative haemoglobin in the two groups was 9.5 gm% and 9.2 gm% respectively (p=0.36) (Fig. 1). This difference was not statistically significant (Table II).

**Table II:** Preoperative and postoperative haemoglobin in both groups

Haemoglobin	Group	N	Mean	SD	p-value
Preoperative	Intervention	67	10.9	2.2	0.79
	Control	70	10.8	2.1	
Postoperative	Intervention	67	9.5	1.8	0.36
	Control	70	9.2	2.0	

**Fig. 2:**
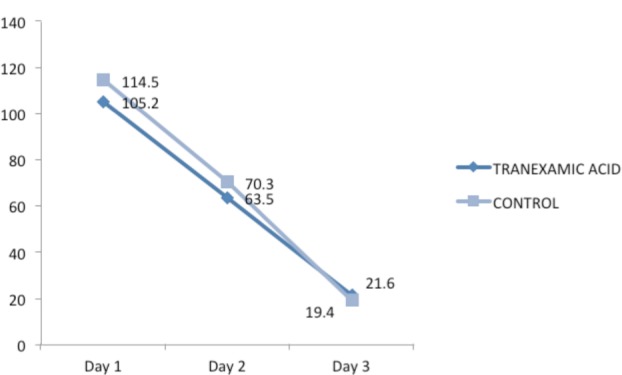
Postoperative blood loss (drain output) in control and trial groups on each of the postoperative days.

The drain was removed on the third postoperative day once the output was below 30 ml as per our institutional protocol. Serial drain outputs were noted to serve as an estimate of total postoperative blood loss. The mean drain outputs in the intervention group on first three days were 105.2 ml, 63.5 ml and 19.4 ml respectively. In the control group the drain output was 114.5 ml, 70.3 ml and 19.4 ml respectively. (Table III) (Fig. 2) None of these differences were statistically significant (p>0.05).

**Table III:** Mean drain output on each of the postoperative days

	Group	N	Mean	SD	p-value
Day 1	Tranexamic acid	67	105.2	41.4	0.17
	Control	70	114.5	38.8	
Day 2	Tranexamic acid	67	63.5	21.9	0.055
	Control	70	70.3	19.3	
Day 3	Tranexamic acid	67	21.6	7.6	0.07
	Control	70	19.4	6.8	
Total	Tranexamic acid	67	190.3	70.4	0.25
	Control	70	204.3	72.7	

The mean total postoperative blood loss in the intervention group and control group was 190.3 ml and 204.3 ml respectively. Although the values were slightly less in the intervention group the p-value was 0.25 and not statistically significant.

As per protocol all patients having postoperative haemoglobin less than 9 gm% received one unit of packed cells transfusion. A total of 10 patients (14.9%) in the intervention group and 12 patients (17.1%) in the control group required blood transfusion (Table III). This difference too was not statistically significant.

## Discussions

Tranexamic acid is a competitive inhibitor of plasminogen activation, and at much higher concentrations, a non-competitive inhibitor of plasmin. As a result of inhibition of the fibrinolysis it prevents the clots from being broken down thereby significantly reducing surgical haemorrhage. Fibrinolysis occurs at increased rate after surgical trauma especially in a major surgery like total knee arthroplasty. Fibrinolysis peaks at six hours after surgery and maintains at a high rate for up to 18 hours after surgery. Thus tranexamic acid used in this period can significantly reduce the blood loss^22^. Tranexamic acid has potential risk of thrombosis in pre-disposed individuals. Other rare side effects include orthostatic symptoms, visual disturbances, headache, myoclonus and rash.

In this study, we assessed the effect of local infiltration of tranexamic acid on various parameters related to blood loss like drain output, haemoglobin drop, postoperative blood loss and requirement for blood transfusion during surgeries for fixation of peritrochanteric fractures. We found that all parameters are lower with the use of tranexamic acid as opposed to controls; however this difference was not statistically significant.

Tranexamic acid has been extensively used in joint replacement surgeries including total knee and total hip arthroplasty^6,11^. Clinical effect of intravenous tranexamic acid in reducing blood loss, haemoglobin drop and need for blood transfusion in total knee arthroplasty has been determined in various studies^6-10^. Efficacy of tranexamic acid in spine surgery also has been proven in many studies. The local administration of tranexamic acid has proven to be extremely effective in knee arthroplasty for reducing blood loss. As a matter of fact local administration is considered to be more effective than intravenous infusion as it is associated with increased efficacy and lesser side effects^20,21^.

Intertrochanteric fractures generally occur in elderly population. Such individuals are more likely to undergo cardiovascular decompensation even with minor blood loss^3^. Although our study revealed consistently lower drain output, haemoglobin drop and requirement for blood transfusion the differences were not significant compared to the control group. None of the patients in the intervention group suffered from any thromboembolic episode after surgery. We suppose that systemic infusion of tranexamic acid could derive additional benefit and local infiltration alone may not be sufficient in making a significant difference.

Only one study has been carried on similar lines in the past. Drakos *et al* in 2016 conducted a randomized prospective trial in 200 intertrochanteric fractures treated with intramedullary nail^23^. The patients in the trial group received 3g of tranexamic acid in the subfascial plane and around the fracture site. They found 43% reduction in transfusion requirements (p<0.01). This is in contrast to our study which did not find any significant differences between the two groups. These differences could be due to the mode of injection and the dosage of the drug. We firmly believe that it is futile to infiltrate tranexamic acid around the fracture site as reduction is achieved by indirect means and the main source of bleeding is in the subfascial and muscular planes. Also, we used dynamic hip screw with barrel plate in all our cases as opposed to nailing done by Drakos *et al.*This surgery involves minimal soft tissue dissection and could have possibly accounted for the difference.

Although our study is prospective, it has certain limitations. Our study could not be double blinded and hence clinician bias could have been introduced. The surgeries were not performed by a single surgeon, thereby leading to differential expertise amongst different surgeons. We looked for the side effects of tranexamic acid only up to two weeks when the patient reported for suture removal. Any potential complications that could have presented later have not been accounted for. We could also not compare our results to the use of isolated intravenous or combined intravenous and local tranexamic acid. This would have helped to conclusively elucidate the role of tranexamic acid in fracture surgery around the hip. A larger randomized controlled trial might be needed to further assess the role of tranexamic acid in hip surgery.

## Conclusion

We conclude that tranexamic acid does not play a significant role in reducing postoperative blood loss and blood transfusion when used locally in peritochanteric fracture surgery. However a larger double blinded study comparing various modalities of use of tranexamic acid is needed to conclusively establish its role.
